# Supplementary material for: Contribution of the Locus of Heat Resistance to Growth and Survival of Escherichia coli at Alkaline pH and at Alkaline pH in the Presence of Chlorine
Source: Microorganisms. 2021 Mar 28;9(4):701. doi: 10.3390/microorganisms9040701 (PMC8067161; doi:10.3390/microorganisms9040701)
Supplement: Supplementary file 1 [file microorganisms-09-00701-s001.pdf]

Online Supplementary Figure to

Contribution of the Locus of Heat Resistance to Growth and Survival of *Escherichia coli* at Alkaline pH and at Alkaline pH in the Presence of Chlorine

Tongbo Zhu, Zhiying Wang, Lynn M. McMullen, Tracy Raivio, David J. Simpson and Michael G. Gänzle

Figure S1. Reduction of viable cell counts of *E. coli* MG1655 and MG1655 *lacZ*::LHR and derivative mutant strains at different pH-values

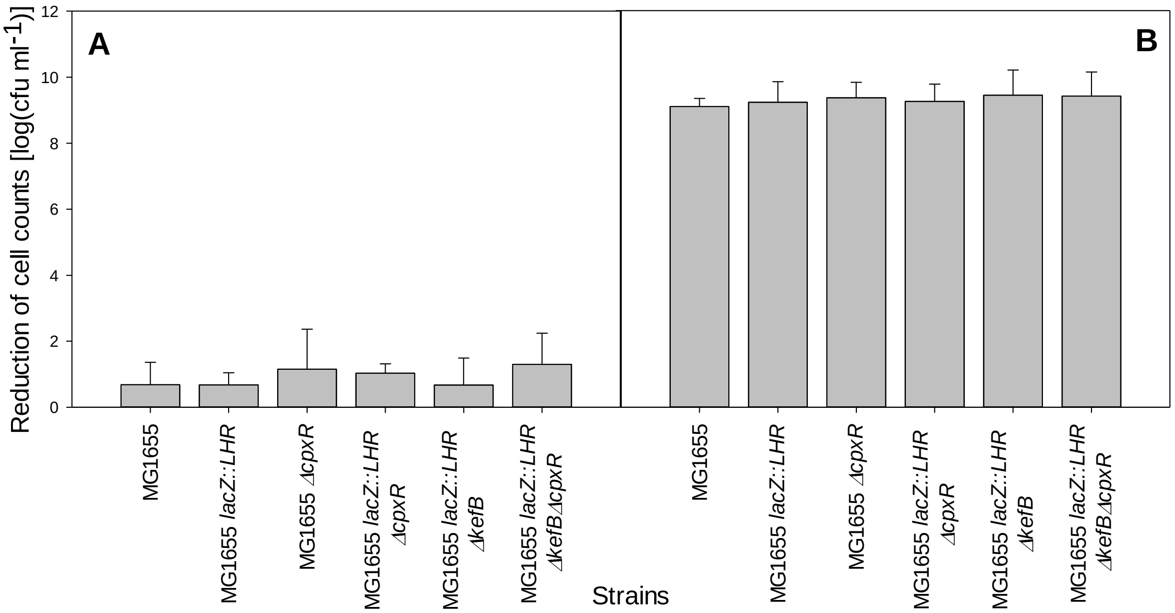

Figure S1. Reduction of viable cell counts of *E. coli* MG1655 and MG1655 *lacZ*::LHR and derivative mutant strains at different pH-values. (**A**) Overnight cultures of all the tested strains were treated with 50mM carbonate-bicarbonate buffered LB at pH 11.0 for 5 min. (**B**) The overnight cultures of all strains were treated at pH 11.3 for 5 min. The bars represent the mean values with standard deviations of three independent experiments. Statistical analysis of the cell count reductions was performed by one-way ANOVA followed by Tukey's HSD; values in the same panel were not different ($P>0.05$).
